# LRSSLMDA: Laplacian Regularized Sparse Subspace Learning for MiRNA-Disease Association prediction

**DOI:** 10.1371/journal.pcbi.1005912

**Published:** 2017-12-18

**Authors:** Xing Chen, Li Huang

**Affiliations:** 1 School of Information and Control Engineering, China University of Mining and Technology, Xuzhou, China; 2 Business Analytics Centre, National University of Singapore, Singapore; University of Calgary Cumming School of Medicine, CANADA

## Abstract

Predicting novel microRNA (miRNA)-disease associations is clinically significant due to miRNAs’ potential roles of diagnostic biomarkers and therapeutic targets for various human diseases. Previous studies have demonstrated the viability of utilizing different types of biological data to computationally infer new disease-related miRNAs. Yet researchers face the challenge of how to effectively integrate diverse datasets and make reliable predictions. In this study, we presented a computational model named Laplacian Regularized Sparse Subspace Learning for MiRNA-Disease Association prediction (LRSSLMDA), which projected miRNAs/diseases’ statistical feature profile and graph theoretical feature profile to a common subspace. It used Laplacian regularization to preserve the local structures of the training data and a *L*_1_-norm constraint to select important miRNA/disease features for prediction. The strength of dimensionality reduction enabled the model to be easily extended to much higher dimensional datasets than those exploited in this study. Experimental results showed that LRSSLMDA outperformed ten previous models: the AUC of 0.9178 in global leave-one-out cross validation (LOOCV) and the AUC of 0.8418 in local LOOCV indicated the model’s superior prediction accuracy; and the average AUC of 0.9181+/-0.0004 in 5-fold cross validation justified its accuracy and stability. In addition, three types of case studies further demonstrated its predictive power. Potential miRNAs related to Colon Neoplasms, Lymphoma, Kidney Neoplasms, Esophageal Neoplasms and Breast Neoplasms were predicted by LRSSLMDA. Respectively, 98%, 88%, 96%, 98% and 98% out of the top 50 predictions were validated by experimental evidences. Therefore, we conclude that LRSSLMDA would be a valuable computational tool for miRNA-disease association prediction.

## Author summary

Discovering miRNA-disease associations promotes the understanding towards the molecular mechanisms of various human diseases at the miRNA level, and contributes to the development of diagnostic biomarkers and treatment tools for diseases. Computational models can make the discovery more efficient and experiments more productive. LRSSLMDA was proposed to computationally infer potential miRNA-disease associations via adopting sparse subspace learning with Laplacian regularization on the known miRNA-disease association network and the informative feature profiles extracted from the integrated miRNA/disease similarity networks. Experimental results in global and local leave-one-out cross validation and 5-fold cross validation showed a superior prediction performance of LRSSLMDA over previous models. Moreover, three types of case studies on five important human diseases were carried out to further demonstrate the model’s predictive power: respectively, 98%, 88%, 96%, 98% and 98% out of the top 50 predicted miRNAs were confirmed by experimental literatures. So, we believe that LRSSLMDA could make reliable predictions and might guide future experimental studies on miRNA-disease associations.

This is a *PLOS Computational Biology* Methods paper.

## Introduction

MicroRNAs (miRNAs) are small (about 22 nucleotides) non-coding RNAs that regulate gene expression [1]. They normally cleave or translationally repress their target messenger RNAs (mRNAs) via base-pairing to the 3’ untranslated region (UTR) sites of the mRNAs [2–5], thereby influencing various biological processes including cell proliferation, development, differentiation, death, apoptosis, metabolism, aging, signal transduction and viral infection [3,6–11]. In addition, increasing studies have indicated a correlation between miRNAs and human diseases [12–19]. For example, the expression level of miR-195 is lowered in Alzheimer’s disease (AD) patients and the AD amyloid-β production could be downregulated by over-expressing this miRNA [20]. Another miRNA mir-26a contributes to the migration of Lung Neoplasms (LN) cells through modulating the expression of metastasis-related genes and suppressing phosphatase and tensin homolog (PTEN) to activate the Protein Kinase B (AKT) pathway [21]. In contrast, miR-145 is under-expressed in LN patients and its restoration inhibits the LN cell proliferation by targeting the EGFR and NUDT1 genes [22]. A further example of miRNA-disease association is miR-501 in Hepatitis B viruses (HBV). Knockdown of this miRNA in the HBV-producing cell line HepG2.2.15 could significantly reduce HBV replication [23]. These miRNAs and many other disease-associated ones may serve as biomarkers for disease diagnosis, progression, prognosis and treatment response [24–27]. Thus, identifying miRNA-disease associations promotes the understanding of complex human diseases and benefits disease treatment. Experimental methods such as microarray profiling and qRTPCR have been used to discover miRNA-disease associations [28]. But they suffer from false-positive microarray results [25,28–30] and are time-consuming and expensive, especially due to the high probe design cost [28]. Fortunately, the large amount of biological data enables researches to develop computational models for predicting disease-related miRNAs. The potential miRNAs are prioritized in terms of prediction scores and the most promising ones are selected for biological verification. This approach complements experimental methods, improving the accuracy of association identification and reducing time and cost.

Remarkable progresses have been achieved in developing prediction models for potential disease-miRNA associations in the past. Most models were based on the assumption that miRNAs with similar functions tend to be associated with phenotypically similar diseases [31–33]. Many previous models were based on network analysis algorithms. An early model for predicting disease-related miRNAs was devised by Jiang *et al*. [34] and it integrated the miRNA functional similarity network, the disease phenotype similarity network and the known disease-miRNA association network. The potential miRNA-disease associations were scored according to a discrete hypergeometric probability distribution. However, the model only considered each miRNA’s neighbor information rather than global similarity measures. Then, Chen *et al*. [35] proposed RWRMDA where novel miRNA-disease associations were predicted by implementing random walking with restart on the miRNA functional similarity network. Although the model achieved an improved prediction accuracy compared with previous models, it was unable to prioritize miRNAs for diseases without any known related miRNAs. Later, Xuan *et al*. [28] developed HDMP, a model that integrated the known miRNA-disease associations and the miRNA functional similarity calculated by incorporating the information content of disease terms and phenotype similarity between diseases. When scoring miRNA-disease pairs, the model included the information of each miRNA’s *k* most similar neighbors and assigned higher weights to miRNAs within the same cluster or family. However, HDMP faced the same problem of failing to predict potential miRNAs related to new diseases without any known associated miRNAs. Subsequently, Shi *et al*. [36] devised another random walk model with a focus on the functional link between miRNA targets and disease genes in a protein-protein interaction (PPI) network. In addition, miRNA-disease co-regulated modules were identified via a hierarchical clustering analysis of a bipartite miRNA-disease network. Nonetheless, involving known disease-gene associations and miRNA-target interactions in the computation impaired the model’s prediction accuracy, since 60% of human diseases have unknown molecular bases [37] and the miRNA-target interactions contain a high rate of false-positive and high false-negative results [35]. Mork *et al*. [38] used a protein-driven approach named miRPD to infer miRNA-protein-disease associations. The model provided not only the potential associations between miRNAs and diseases but also the protein links between them. To make the inference, known and predicted protein-miRNA interactions were coupled with protein-disease associations text-mined from experimental literatures. Then the inferred miRNA-protein-disease associations were ranked by confidence under two scoring schemes; and the ranking results were divided into a high-confidence subset holding the most probable associations and a medium-confidence subset including the less likely associations. Xuan *et al*. [39] further introduced a random walk model named MIDP that exploited the prior information of nodes and various ranges of topologies in a miRNA-disease bilayer network derived from the miRNA functional similarity network, the disease semantic similarity network, and the edges between the two networks. With an extended walk on the network, the model overcame the limitations of previous models and could make association predictions for diseases that has no known related miRNAs. Furthermore, the negative effect of noisy data was mitigated via adjusting the restart rate of the random walk. To improve the prediction accuracy, Chen *et al*. [40] released WBSMDA that calculated and combined the within and between scores from the views of miRNAs and diseases in a composite network, built from the known miRNA-disease associations, the miRNA functional similarity, the disease semantic similarity and the Gaussian interaction profile kernel similarity networks for diseases and miRNAs. Gu *et al*. [41] developed a non-parametric universal network-based model named NCPMDA. In this model, a miRNA similarity network was constructed by combining the miRNA functional similarity, the Jaccard miRNA similarity of the known miRNA-disease associations and the miRNA family information; and a disease similarity network was built by integrating the disease semantic similarity and the Jaccard disease similarity of the known associations. Then, network consistency projection was carried out on the miRNA similarity network to the adjacency matrix of miRNA-disease associations, and on the disease similarity network to the adjacency matrix, respectively. Lastly, the miRNA space projection scores and the disease space projection scores were combined and normalized to give the final prediction scores. Chen et al. [42] further presented HGIMDA in which a heterogeneous graph network was constructed using the same model inputs as WBSMDA. Then, an iterative process was carried out in the network until a stable association probability matrix was obtained. Following HGIMDA, MCMDA was published by Li *et al*. [43] utilizing a matrix completion algorithm on the low-rank miRNA-disease association matrix. The candidate miRNA-disease pairs in the matrix were iteratively updated with predictive association scores, yielding highly reliable outcomes. Yu *et al*. [44] proposed a combinatorial prioritization algorithm named MaxFlow. The model’s input included the miRNA functional similarity network, the disease semantic and phenotypic similarity network, and the heterogeneous miRNA-disease association network that integrated miRNA-disease associations, the miRNA family information and the miRNA cluster information. Subsequently, these three networks were further combined to form a directed miRNAome-phenome network graph, where the weight of each link was regarded as the flow capacity. For an investigated disease, a source node and a sink node were introduced to this graph; and the maximum information flow from the source over all links to the sink were calculated using the push-relabel maximum flow algorithm. The flow quantity leaving a miRNA node was used as the association score between the miRNA and the investigated disease. More recently, You *et al*. [45] devised path-based model named PBMDA, where a heterogeneous graph were built from the same input datasets as those in WBSMDA. In the graph, all paths between a miRNA-disease pair were traversed via the adoption of the depth-first search algorithm; and each path’s score was computed by multiplying all the edges’ weights along the path. For a longer path, the score would be penalized by a distance-decay function. The sum of scores for all the paths were used as the association score for the miRNA-disease pair.

In addition, other previous models were based on machine learning algorithms. Xu *et al*. [46] used a support vector machine classifier to separate positive and negative miRNA-disease associations in a heterogeneous miRNA-target dysregulated network (MTDN). Negative samples were required to train the model. However, finding negative miRNA-disease associations is a difficult or even impossible task [42], meaning that the prediction accuracy might be reduced because the model is learned from inappropriate training samples. To address this problem, Chen *et al*. [47] applied semi-supervised learning (RLSMDA) to the inference of miRNA-disease associations and only using positive samples would suffice the model-training. The ensuing model was RBMMMDA authored by Chen *et al*. [48]. Restricted Boltzmann machine was implemented to predict four different types of miRNA-disease associations from a two-layered (with visible and hidden units) undirected miRNA-disease graph. RBMMMDA was the first model not only prioritizing potential associations but also providing the corresponding association types. A more recent model developed by Chen *et al*. [49] was ranking-based k-nearest neighbors for miRNA-disease association prediction (RKNNMDA). It was a three-staged approach: initially running the k-nearest neighbors algorithm for miRNAs and diseases, then carrying out SVM Ranking to rank the neighbors and lastly weighted-voting for both miRNAs and diseases to reduce the prediction bias. Later, Pasquier *et al*. [50] introduced a vector space model named MiRAI that formed a large network via concatenating five association networks, namely, the miRNA-disease association network, the miRNA-neighbor association network with edges weighted by the genomic distance between two miRNA nodes, the miRNA-target association network, the miRNA-word association network with edges weighted by the term frequency–inverse document frequency (TF-IDF) information retrieval scheme on investigated miRNAs’ associated documents, and the miRNA-family association network. Then, the large combined network was decomposed by Singular Value Decomposition (SVD) into the form of *UΣV*^*T*^, where the columns of *U* were the left-singular vectors, *Σ* was the matrix of nonnegative real numbers on the diagonal, and the columns of *V* were the right-singular vectors. The association score for a miRNA-disease pair was calculated by the cosine similarity between the vector of the miRNA in the miRNA space (*U*) and the vector of the disease in the disease space (a part of *V*).

The above mentioned models had their own strengths and uniqueness, while several of them suffered from obvious weaknesses. More importantly, although most models exhibited a sound prediction accuracy, there still exist areas for a continued improvement. When informative feature profiles were extracted from the training data, the challenge would be how to achieve a single classifier that reasonably combine multiple profile spaces. Hence in this study we presented a model of Laplacian Regularized Sparse Subspace Learning for MiRNA-Disease Association prediction (LRSSLMDA) to meet the challenge. The Gaussian interaction profile kernel similarity for miRNA and diseases was computed and integrated with the miRNA functional similarity and the disease semantic similarity. Although the Gaussian interaction profile kernel similarity had been successfully used by Chen *et al*. [51] in the LRLSLDA model for lncRNA-disease association prediction, their data preparation process was different from that in our study. For LRLSLDA, data preparation involved the lncRNA expression similarity and the lncRNA-disease associations; and the disease semantic similarity was not used. The Gaussian interaction profile kernel similarity for diseases and lncRNAs were computed from the lncRNA-disease associations. Then, the disease similarity was calculated by performing logistic function transformation on the Gaussian interaction profile kernel similarity for diseases; and the integrated similarity for lncRNAs was built by combining the Gaussian interaction profile kernel similarity for lncRNAs and the lncRNA expression similarity. Moreover, a weight coefficient was used in the integrated similarity for lncRNAs. From this, it is apparent that our model and LRLSLDA had different data preparation processes. In addition, constructing the integrated similarity for diseases and miRNAs was only the first step of our model’s data preparation. As the ensuing and important step, feature extraction was performed on the integrated similarity to form the statistical profile and the graph theoretical profile, and these two informative feature profiles were a key to the success of LRSSLMDA. Subsequently, the model used sparse subspace learning to map high dimensional miRNA/disease spaces into a lower dimensional subspace; and it used Laplacian regularization to smooth the subspace and maintain the local structures of the high dimensional spaces. The combination of these two techniques has been successfully applied to web image categorization by Shi *et al*.’s [52] and drug-target interaction prediction by Liang *et al*. [53]. But different from Liang *et al*.’s model, our model made effective predictions with fewer input datasets, exploited informative disease-related feature profiles, and could be applied to diseases without known associations. LRSSLMDA achieved effective dimensionality reduction and could simultaneously analyze a large amount of unlabeled data and a small amount of labeled data. The model was evaluated in three cross validation schemes and three types of case studies on five diseases. In local leave-one-out cross validation (LOOCV), global LOOCV and 5-fold cross validation, LRSSLMDA outperformed ten previous models; and for each disease in case studies, our model predicted the top 50 potentially associated miRNAs and most of the predictions were confirmed by experimental literatures.

## Materials and methods

### Human miRNA-disease associations

HMDD v2.0 is a human miRNA-disease association database that records 5430 experimentally supported associations between 495 miRNAs and 383 diseases (See S2 Table). We used *nm* to denote the number of miRNAs, *nd* for the number of diseases and *MDA* for the *nm* × *nd* adjacency matrix made up of the *nm* miRNAs and the *nd* diseases. If miRNA *m*(*i*) had a known association to disease *d*(*j*), the entity *MDA*(*m*(*i*), *d*(*j*)) would equal to 1, and otherwise 0.

### MiRNA functional similarity

MiRNA functional similarity scores used in our study were retrieved from http://www.cuilab.cn/files/images/cuilab/misim.zip and computed based on the hypothesis that miRNAs with a functional similarity are more likely to correlate with diseases with a phenotypical similarity [54]. A *nm* × *nm* miRNA functional similarity network *FS* was constructed with weighted edges. An entity *FS*(*m*(*i*), *m*(*j*)) denoted the functional similarity score between miRNA *m*(*i*) and *m*(*j*).

### Disease semantic similarity

As illustrated in the literature [28], the semantic information of disease *d*(*i*) was explained by a Directed Acyclic Graph (DAG) where *d*(*i*) and its ancestor diseases were used as nodes. The DAGs were retrieved from the U.S. National Library of Medicine (MeSH) at https://www.nlm.nih.gov/mesh/. The relationship between a parent node and a child node was represented by a directed edge pointing from the former to the latter. For disease *t* in DAG(*d*(*i*)), its contribution to the semantic value of *d*(*i*) was computed by

$$D_{d\left(i\right)}\left(t\right)\;=\;-\text{log}\left(\frac{\text{the}\;\text{number}\;\text{of}\;\text{DAGs}\;\text{including}\;t}{\text{the}\;\text{number}\;\text{of}\;\text{diseases}}\right)$$ (1)

The rationale behind (1) was that a greater contribution should be made by a more specific disease *t* to the semantic value of *d*(*i*). Summing up all the contributions from *d*(*i*)’s ancestor diseases and itself gave its semantic value

$$DV\left(d\left(i\right)\right)=\sum_{t\in D\left(d\left(i\right)\right)}D_{d\left(i\right)}\left(t\right)$$ (2)

where *D*(*d*(*i*)) denoted the node set in DAG(*d*(*i*)). Subsequently, the semantic similarity between disease *d*(*i*) and *d*(*j*) was defined by:

SS(d(i),d(j))=∑t∈D(d(i))∩D(d(j))(Dd(i)(t)+Dd(j)(t))DV(d(i))+DV(d(j)) (3)

This equation implied that two diseases with a greater overlap of their DAGs would exhibit a higher semantic similarity score between them.

### Gaussian interaction profile kernel similarity for miRNAs

According to [55], the Gaussian kernel similarity between miRNA *m*(*i*) and miRNA *m*(*j*) was calculated as follows. Respectively, binary interaction profile vectors *IP*(*m*(*i*)) and *IP*(*m*(*j*)) were used to represent the *i*th column and the *jth* column of *MDA* and were then fed into the Gaussian interaction profile kernel similarity matrix for miRNAs, *KM*

$$\begin{array}{c} KM\left(m\left(i\right),\;m\left(j\right)\right)\;=\;\text{exp}\;\left(-\gamma_{m}∥IP\left(m\left(i\right)\right)-IP\left(m\left(j\right)\right){∥}^{2}\right) \end{array}$$ (4)

where *γ*_*m*_ was the bandwidth parameter for the function. It was defined by another parameter *γ*′_*m*_ and the average number of associated diseases for all miRNAs

γm=γ’m1nm∑i=1nm∥IP(m(i))∥2 (5)

Same to previous literatures [51,55], both the values of *γ*_*m*_ and *γ*′_*m*_ were set to 1 for the simplicity of calculations.

### Gaussian interaction profile kernel similarity for diseases

Similar to miRNAs, the diseases’ Gaussian interaction profile kernel similarity matrix *KD* was calculated by

KD(d(i),d(j))=exp(-γd∥IP(d(i))-IP(d(j))∥2) (6)

where binary interaction profile vectors *IP*(*d*(*i*)) and *IP*(*d*(*j*)) denoted the *i*th row and the *jth* row of *MDA*; and *γ*_*d*_ was the bandwidth parameter defined by another parameter *γ*′_*d*_ and the average number of associated miRNAs for all diseases

γd=γ’d1nd∑i=1nd∥IP(d(i))2∥ (7)

Again, as with the literatures [51,55], in our study we set the values of *γ*_*d*_ and *γ*′_*d*_ to 1 to make the calculations simple.

### Integrated miRNA similarity and diseases

The miRNA functional similarity matrix *FS* and the Gaussian interaction profile kernel similarity matrix *KM* were integrated to form a more comprehensive similarity measure, which was the integrated similarity matrix for miRNAs *SM*

SM(m(i),m(j))={FS(m(i),m(j)),ifm(i)andm(j)havefunctionalsimilarityKM(m(i),m(j)),otherwise (8)

This means that if miRNA *m*(*i*) and *m*(*j*) had a functional similarity, we chose their corresponding score in *FS* to be their integrated similarity score; otherwise, we chose instead their Gaussian kernel similarity score obtained from (4).

Similarly, the disease integrated similarity matrix *SD* was obtained from the disease semantic similarity matrix *SS* and the Gaussian interaction profile kernel similarity matrix *KD*

SD(d(i),d(j))={SS(d(i),d(j)),ifd(i)andd(j)havesemanticsimilarityKD(d(i),d(j)),otherwise (9)

### LRSSLMDA

**Fig 1:**
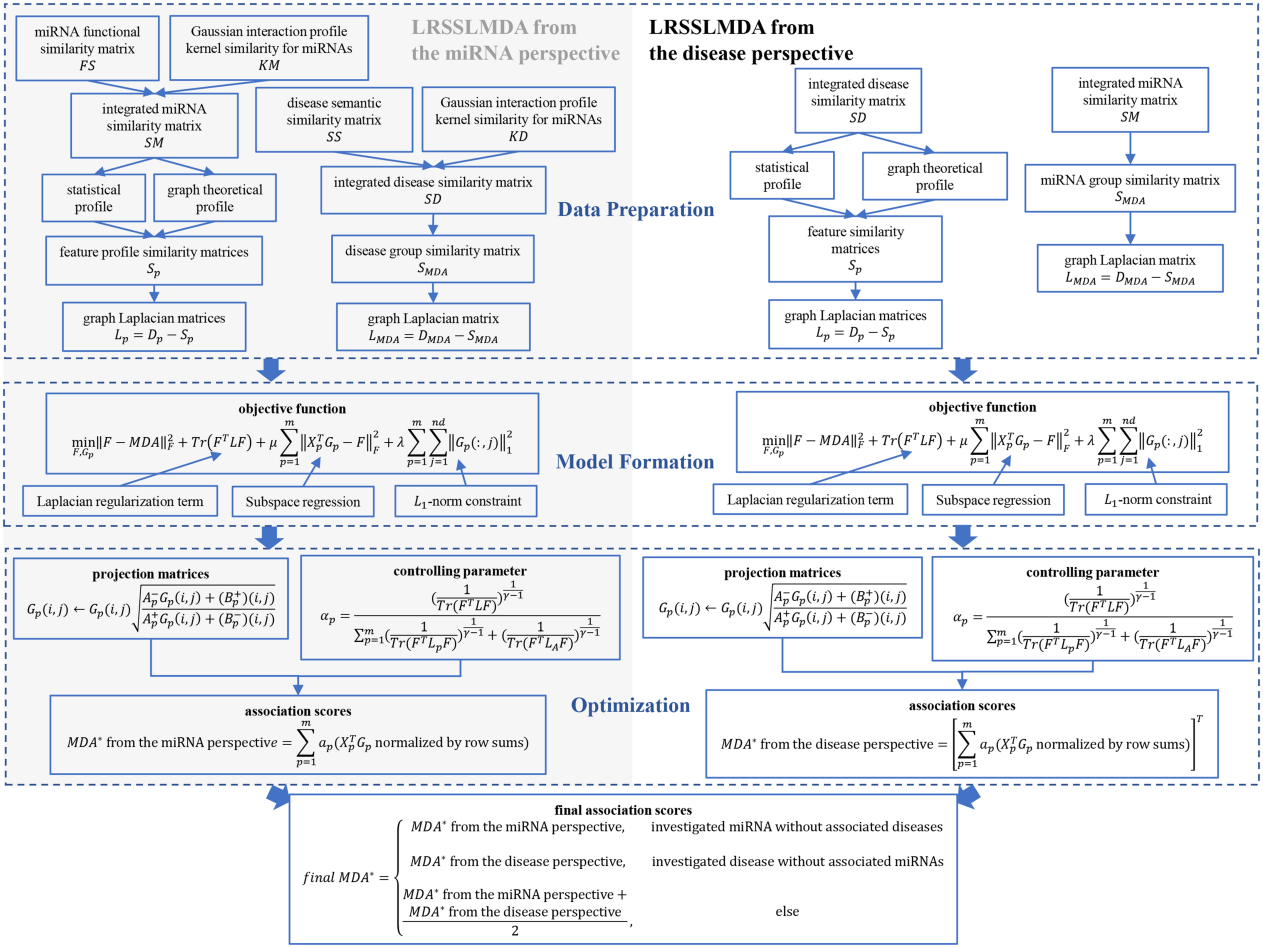
Flowchart of potential miRNA-disease association prediction based on the computational model of LRSSLMDA. 1) data preparation, where statistical and graph theoretical features for miRNAs/diseases were extracted and graph Laplacian matrices were formed; 2) model formation, where a common subspace for the miRNA/disease profiles, a *L*_*1*_-norm constraint and Laplacian regularization terms were joint to construct the LRSSLMDA model; 3) optimization, where the projection matrices were iteratively updated, the controlling parameter was renewed and they were combined to yield the prediction outcomes from the miRNA/disease perspective. The final predictions were made according to whether the investigated miRNA/disease had known associated diseases/miRNAs or not.

In this study, we developed LRSSLMDA to uncover potential miRNA-disease associations. The model inputs included the miRNA-disease association matrix *MDA*, the miRNA functional similarity matrix *FS* and the disease semantic similarity matrix *SS*. The procedure of implementing LRSSLMDA involved data preparation, model formulation and optimization, as depicted in Fig 1. In data preparation, the integrated similarity matrices *SM*/SD were constructed according to (8) and (9), respectively, before being used to form two types of feature profiles for miRNAs/diseases. The idea of performing feature extraction on similarity networks to obtain feature profiles originated from the literature [56]. In our study, the first type of profile summarized *SM*/SD from a statistical perspective, so it was known as the statistical profile. For miRNA *m*(*i*)/disease *d*(*j*), we calculated

- *n*.*obs*, the number of observed associations in the corresponding *i*th row/*j*th column of *MDA*, namely, the sum of the *i*th row/*j*th column of *MDA* for miRNA *m*(*i*)/disease *d*(*j*). The rationale for using this metric was as follows. When making predictions for a specific miRNA *m*(*i*)/disease *d*(*j*), our method would analyze not only *m*(*i*)/*d*(*j*)’s known associated diseases/miRNAs but also these diseases/miRNAs’ similar diseases/miRNAs. The more known associated diseases/miRNAs *m*(*i*)/*d*(*j*) had, the more data would be analyzed to support the predictions for *m*(*i*)/*d*(*j*). Therefore, a higher value of *n*.*obs* for a miRNA/disease indicated that more reliable predictions would likely be made for the miRNA/disease.
- *ave*.*sim*, the average of similarity scores for miRNA *m*(*i*)/disease *d*(*j*), namely, the average of the *i*th/*j*th row of *SM*/SD
- *s*.*d*.*sim*, the standard deviation of similarity scores for miRNA *m*(*i*)/disease *d*(*j*)
- *min*.*sim*, the minimum of similarity scores for miRNA *m*(*i*)/disease *d*(*j*)
- *first*.*q*.*sim*, the first quantile value of similarity scores for miRNA *m*(*i*) /disease *d*(*j*)
- *median*.*sim*, the median of similarity scores for miRNA *m*(*i*) /disease *d*(*j*)
- *third*.*q*.*sim*, the third quantile value of similarity scores for miRNA *m*(*i*) /disease *d*(*j*)
- *max*.*sim*, the maximum of similarity scores for miRNA *m*(*i*) /disease *d*(*j*)
- *hist*.*sim*, the histogram feature; the range of similarity scores [0, 1] was segmented into *n* bins (*n* equaled 10 in this study) and we counted the proportion of similarity scores for *m*(*i*)/*d*(*j*) that fell into each bin

The second type of profile described *SM*/SD using graph theories, hence was named graph theoretical profiles. We converted *SM*/SD into an unweighted graph version: miRNA *m*(*i*)/disease *d*(*j*) now became a node in the graph; and an edge would form between two nodes if their similarity score surpassed the mean value of all entities. For each node in the unweighted graph version of *SM*/SD, we calculated

- *num*.*nb*, the number of neighbors of the node
- *k*.*sim*, the similarity values of the *k*-nearest neighbors of the node (*k* equaled 10 in this study, so this was a vector of 10 elements)
- *k*.*ave*.*feat*, the average of statistical features (defined in the statistical profile) for the *k*-nearest neighbors of the node
- *k*.*w*.*ave*.*feat*, the average of statistical features for the *k*-nearest neighbors of the node weighted by the neighbors’ similarity scores
- *bt*,*cl*,*ev*, the betweenness, closeness, eigenvector centralities of the node
- *pr*, the Page-Rank score of the node

Inspired by Liang *et al*.’s LRSSL model for drug-disease association prediction [53], we used the feature profiles for miRNAs and diseases separately to form and optimize two respective LRSSLMDA objective functions. Our model was an innovation to Liang *et al*.’s model in the following aspects. First, LRSSLMDA could make effective predictions with fewer input datasets than Liang *et al*.’s model. As aforementioned, the input to our model contained only three datasets, namely, the miRNA functional similarity, the disease semantic similarity and the known miRNA-disease associations. On the other hand, their model predicted associations between drugs and diseases by integrating five datasets: the drugs’ chemical substructure profile, the drugs’ target protein domain profile, the drugs’ gene ontology term profile, the disease semantic similarity and the known drug-diseases associations. Second, Liang *et al*.’s model was developed mainly based on the ready-made drug-related profiles and so was only able to work from the drug perspective. Liang *et al*.’s literature stated that a limitation of the method was not being able to exploit disease-related profiles. Without the involvement of disease-related profiles, the model could not achieve the best possible performance. To deal with this limitation, we made the most of the available disease information by constructing the integrated disease similarity, extracting the statistical profile and the graph theoretical profile for diseases from the integrated similarity, and building the objective function from the disease perspective. In this manner, our model could accurately infer miRNA-disease associations. Third, by intensively involving disease feature profiles, our model could be applied to diseases without known associated miRNAs, whereas Liang *et al*.’s model was not effective in uncovering drugs associated with a disease that had no known associated drugs.

Because the two objective functions from the miRNA and disease perspectives were constructed and optimized in a similar manner, the rest of this section elaborates the remaining data preparation step, the model formation step and the optimization step from the view of miRNAs, while briefly presenting these steps from the view of diseases.

For miRNAs, the two feature profiles were represented by *X*_*p*_ where *p* equaled 1, 2 to denote the first and second profiles; the dimension of *X*_*p*_ was *d*_*p*_ × *nm* where *d*_*p*_ was the number of features for the *p*th profile. For each profile, we further built a network graph *S*_*p*_, whose elements were defined by

Sp(i,j)={1,ifXp(j)wasthek-nearestneighborofXp(i)0,otherwise (10)

where *X*_*p*_(*i*) and *X*_*p*_(*j*) were respectively the *i*th and *j*th vectors of the *p*th feature profile. Their closeness was measured by the cosine similarity between them. Furthermore, for miRNAs with known related diseases, we constructed another network graph *S*_*MDA*_, whose elements were computed by

SMDA(i,j)={1,ifMDA(m(j))wasthek-nearestneighborofMDA(m(i))0,otherwise (11)

where *MDA*(*m*(*i*)) and *MDA*(*m*(*j*)) were respectively the *i*th and *j*th row of *MDA*. Their closeness equaled the maximum integrated similarity score between *m*(*i*) and *m*(*j*)’s associated disease groups. The last part of the data preparation step was to construct graph Laplacian matrices *L*_*p*_ and *L*_*A*_

Lp=Dp-Sp (12)

where *D*_*p*_ was the diagonal matrix of *S*_*p*_ in the form of

$$D_{p}\left(i,i\right)=\sum_{j}^{n}S_{p}\left(i,j\right)$$ (13)

Similarly,

LMDA=DMDA-SMDA (14)

where *D*_*MDA*_ was the diagonal matrix of *S*_*MDA*_ and defined by

$$D_{MDA}\left(i,i\right)=\sum_{j}^{n}S_{MDA}\left(i,j\right)$$ (15)

*L*_*p*_ and *L*_*MDA*_ were used to form a Laplacian regularization term in our model and to smooth a subspace to which the miRNA profiles were projected. *L*_*p*_ reflected the trend that miRNAs with similar features should be related to similar diseases, while *L*_*MDA*_ helped to maintain the similarity between different miRNAs’ related disease groups.

The subsequent step was model formation, where a common subspace for the miRNA profiles, a *L*_*1*_-norm constraint and Laplacian regularization terms were joint to construct the LRSSLMDA model. This formation was consistent with that presented in the literature [53] and conveyed as the objective function below. This function effectively projected the miRNA profiles to a common subspace and maintained both the local and global structure of the input data.

$$\begin{array}{c} \underset{F,G_{p}}{\text{min}}∥F-MDA{∥}_{F}^{2}+Tr\left(F^{T}LF\right)+\mu \sum_{p=1}^{m}∥X_{p}^{T}G_{p}-F{∥}_{F}^{2}\\ +\lambda \sum_{p=1}^{m}\sum_{j=1}^{nd}∥G_{p}\left(:,j\right){∥}_{1}^{2} \end{array}$$ (16)

$$\text{s}\text{.t}\text{.}\;G_{p}\geq 0$$

In (16), *F* was the predicted miRNA-disease association matrix. The first term ${∥F-MDA∥}_{F}^{2}$ was to keep *F* aligned with *MDA*, and ||·||_*F*_ was the Frobenius norm.

*Tr*(*F*^*T*^*LF*) was the Laplacian regularization term, where $L\;=\;\sum_{p\;=\;1}^{m}\alpha_{p}^{\gamma }L+\alpha_{MDA}^{\gamma }L_{MDA}$. Here, *α* controlled the contribution of different graph Laplacian matrices to the predictions and γ > 1 guaranteed that all graph Laplacian matrices made a contribution. *m* was the number of miRNA feature profiles and equaled 2 in this study.

$\mu \sum_{p\;=\;1}^{m}{∥X_{p}^{T}G_{p}-F∥}_{F}^{2}$ was the subspace regression term, where $X_{p}^{T}G_{p}$ was a common subspace in the form of a linear transformation of *X*_*p*_, and *G*_*p*_ was the projection matrix of the *p*th miRNA feature profile. The subspace was learnt by minimizing the regression errors and *μ* was the balancing parameter for the subspace learning.

$\lambda \sum_{p\;=\;1}^{m}\sum_{j\;=\;1}^{nd}{∥G_{p}(:,j)∥}_{1}^{2}$ was the *L*_*1*_-norm constraint, used to impose sparsity on *G*_*p*_ and assign weights to miRNA features. Here, *λ* was the regularization parameter and *G*_*p*_(:, *j*) was the *j*th column of *G*_*p*_.

Finally, (16) was optimized in an iterative process where *α*_1_, *α*_2_ and *α*_*MDA*_ were initialized to 1/3 and *G*_1_ and *G*_2_ began with random non-negative values from uniform distribution on the [0, 1] interval. According to [53], *γ* was set to 2; and since the algorithm was not that sensitive to the values of *μ* and *λ*, we have set both of them to 1 for the simplicity in calculation. All parameters could be optimized by further cross validation. *G*_*p*_ was interactively updated based on the auxiliary function approach [57]

Gp(i,j)←Gp(i,j)Ap-Gp(i,j)+(Bp+)(i,j)Ap+Gp(i,j)+(Bp-)(i,j (17)

where

Ap=Xp(μI-μ2PT)XpT+λe1×dpTe1×dp (18)

$$B_{p}=\mu X_{p}PY+\mu^{2}\sum_{q\neq p}^{m}X_{p}P^{T}X_{q}^{T}G_{q}$$ (19)

P=(L+(1+mμ)I)-1 (20)

and $e_{1\times d_{p}}$ was a 1×*d*_*p*_ vector with all elements equal to 1. By fixing *F* and *G*_*p*_, *α*_*p*_ was renewed by the equation introduced in [52].

αp=(1Tr(FTLF))1γ-1∑p=1m(1Tr(FTLpF))1γ-1+(1Tr(FTLAF))1γ-1 (21)

The derivation and convergence proof of the optimization algorithm were presented in [53]. The final *G*_*p*_ was multiplied by *X*_*p*_ and then was normalized by row sums, before further timed by the final *α*_*p*_. In this way, the predicted association scores for all miRNA-disease pairs from the view of miRNAs were obtained

$$\begin{array}{c} MDA^{*}\;\text{from}\;\text{the}\;\text{miRNA}\;\text{perspective}\\ =\sum_{p=1}^{m}a_{p}\left(X_{p}^{T}G_{p}\;\text{normalized}\;\text{by}\;\text{row}\;\text{sums}\right) \end{array}$$ (22)

Similarly, for diseases in Data Preparation, the two feature profiles were denoted by *X*_*p*_ where *p* equaled 1, 2 to denote the first and second profiles; the dimension of *X*_*p*_ was *nm* × *d*_*p*_ where *d*_*p*_ was the number of features for the *p*th profile. The resulting network graphs for disease profiles were obtained in the same way as (10). For diseases with known related miRNAs, the network graph *S*_*MDA*_ was given by

SMDA(i,j)={1,ifMDA(d(j))wasthek-nearestneighborofMDA(d(i))0,otherwise (23)

Then graph Laplacian matrices *L*_*p*_ and *L*_*A*_ were calculated according to (12) and (14). Again, we constructed the objective function based on (16) in Model Formation, and the Optimization step gave the predicted association scores for all miRNA-disease pairs from the view of diseases

$$\begin{array}{l} MDA*\text{from}\;\text{the}\;\text{disease}\;\text{perspective}\\ ={\left[\sum_{p=1}^{m}a_{p}\left(X_{p}^{T}G_{p}\;\text{normalized}\;\text{by}\;\text{row}\;\text{sums}\right)\right]}^{T} \end{array}$$ (24)

The final prediction scores for all miRNA-disease pairs were computed according to three scenarios. First, when predicting potential diseases associated with a miRNA that had no associated diseases, the final prediction scores were calculated according to (22) only, which was MDA* from the miRNA perspective. Second, when predicting potential miRNAs associated with a disease that had no associated miRNAs, the final prediction scores were calculated based on (24) only, which was MDA* from the disease perspective. Third, when making predictions for a miRNA/disease with some associated diseases/miRNAs, the final prediction scores were obtained by taking the average of (22) and (24). These three scenarios were depicted as in (25)

$$final\;MDA*=\{\underset{\begin{array}{cc} \frac{\begin{array}{c} MDA*\;\text{from}\;\text{the}\;\text{miRNA}\;\text{perspective}\\ +MDA*\;\text{from}\;\text{the}\;\text{disease}\;\text{perspective} \end{array}}{2}, & \text{else} \end{array}}{\begin{array}{cc} MDA*\;\text{from}\;\text{the}\;\text{miRNA}\;\text{perspective}, & \begin{array}{c} \text{investigated}\;\text{miRNA}\\ \text{without}\;\text{associated}\;\text{diseases} \end{array}\\ MDA*\;\text{from}\;\text{the}\;\text{disease}\;\text{perspective}, & \begin{array}{c} \text{investigated}\;\text{disease}\\ \text{without}\;\text{associated}\;\text{miRNAs} \end{array} \end{array}}$$ (25)

## Results

### Performance evaluation

In this study, we implemented both global and local LOOCV validation methods based on 5430 known miRNA-disease associations between 383 diseases and 495 miRNAs from HMDD v2.0 to evaluate the prediction accuracy of LRSSLMDA. Global LOOCV focused on all potential miRNA-disease associations. Each known miRNA-disease association was left out in turn as the test sample (hence 5430 validation rounds in total), while all the other known associations were considered as the training samples. The remaining miRNA-disease pairs were regarded as candidates. A candidate means a miRNA-disease pair whose association was unconfirmed according to HMDD v2.0 and needed to be predicted by LRSSLMDA. In contrast, local LOOCV only considered miRNAs for a specific disease. Each known miRNA related to disease *d*(*i*) was left out in turn as the test sample. This time, we defined all other known disease-related miRNAs (including those related to diseases other than disease *d*(*i*)) to be the seeds, and the miRNAs under the unconfirmed association status with disease *d*(*i*) to be the candidates. For both global and local LOOCV, the test sample was ranked by LRSSLMDA against the candidates; a rank exceeding a predefined threshold would indicate a successful prediction made by the model and vice versa. Then we plotted a Receiver Operating Characteristics curve with the true positive rate (TPR, sensitivity) versus the false positive rate (FPR, 1-specificity) at various thresholds. Sensitivity meant the percentage of test samples ranked above the threshold and specificity represented the percentage of candidates ranked below the threshold. ROC was subsequently used to generate Area under the ROC curve (AUC), a statistic widely used for describing the prediction accuracy of computational model. An AUC of 1 indicates a perfect performance whereas an AUC of 0.5 implies a random performance.

**Fig 2:**
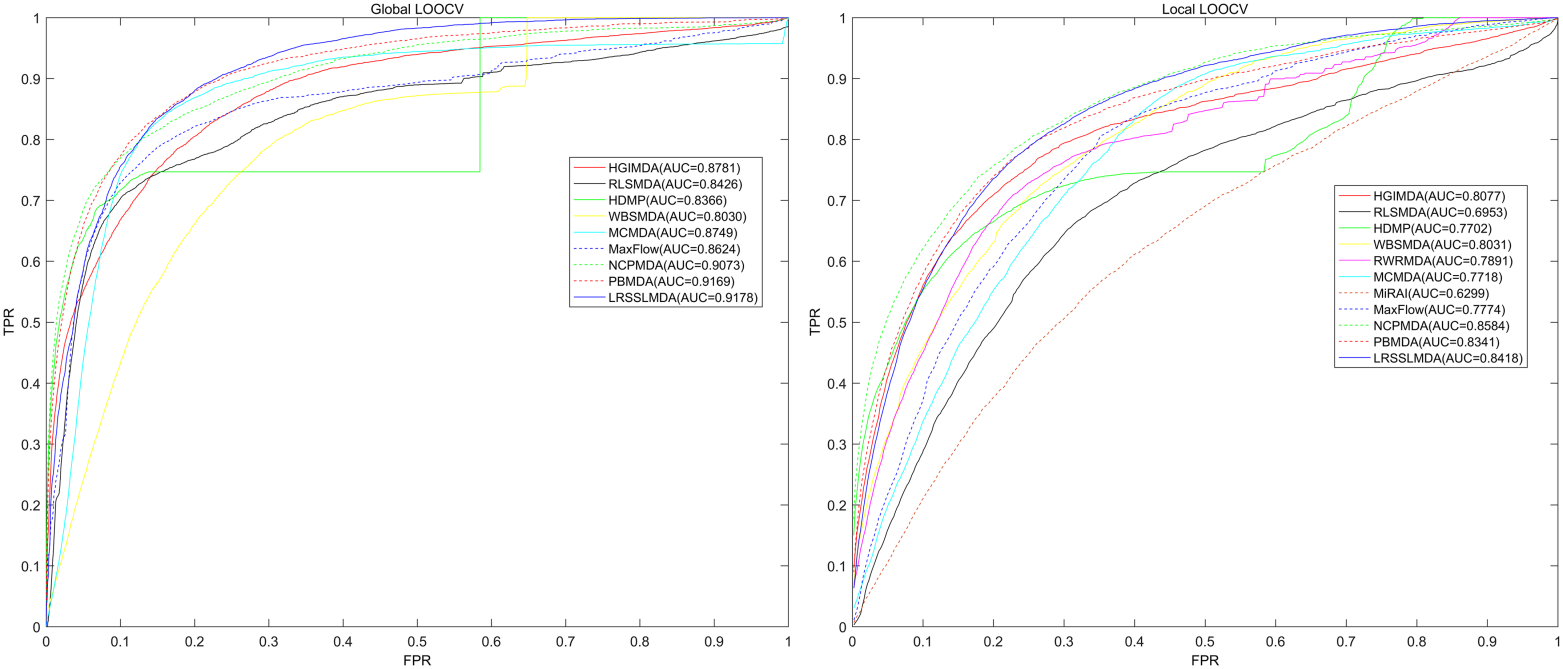
Performance comparison between LRSSLMDA and ten previous disease-miRNA association prediction models (PBMDA, MaxFlow, MCMDA, NCPMDA, HGIMDA, MiRAI, WBSMDA, HDMP, RLSMDA and RWRMDA) in terms of ROC curves and AUCs based on global and local LOOCV. As a result, LRSSLMDA outperformed other models by achieving an AUC of 0.9178 in global LOOCV and an AUC of 0.8418 in local LOOCV.

As shown in Fig 2, in global LOOCV, LRSSLMDA achieved an AUC of 0.9178 and was superior to PBMDA (0.9169), MCMDA (0.8749), MaxFlow (0.8624), NCPMDA (0.9073), HGIMDA (0.8781), WBSMDA (0.8030), HDMP (0.8366) and RLSMDA (0.8426). RWRMDA was not compared in global LOOCV because the model was based on a local ranking approach and thus unable to simultaneously uncover potential miRNAs for all diseases. MiRAI was not implemented in global LOOCV, either. By analyzing association scores calculated by this model, we found that the scores were highly positively correlated with the seed count (i.e., the number of known associated miRNAs) of the investigated disease. We calculated the correlation coefficient between the mean/median score for a disease and the seed count of the disease:

correlation(meanassociationscore,seedcount)=0.4567

correlation(medianassociationscore,seedcount)=0.3979

From this, we can see that the more associated miRNAs a disease had, the higher the association scores for its candidate miRNAs would be; and vice versa. Thus, the association scores calculated by MiRAI for different diseases were not globally comparable and the model was a local method, not applicable to global LOOCV.

In local LOOCV, our model yielded an AUC of 0.8418 and outperformed PBMDA (0.8341), MaxFlow (0.7774), MCMDA (0.7718), HGIMDA (0.8077), MiRAI (0.6299), WBSMDA (0.8031), HDMP (0.7702), RLSMDA (0.6953) and RWRMDA (0.7891). Although our model underperformed NCPMDA (0.8584), the former was superior to the latter both in global LOOCV as mentioned above and in 5-fold cross validation to be subsequently discussed after local LOOCV. Furthermore, NCPMDA seemed sensitive to the percentage of known associations in the training data. In Gu *et al*.’s study [41], the model was evaluated by local LOOCV using the 1395 known associations between 271 miRNAs and 137 diseases in the HMDD v1.0 database; and the resulting AUC was 0.9173, much higher than the value of 0.8584 obtained in our study. This was due to a reduction of the ratio of known associations to all miRNA-disease pairs in the training data: in HMDD v1.0 there were 1395/(271×137) = 3.76% of miRNA-disease pairs known to be associated, whereas in HMDD v2.0 there were 5430/(495×383) = 2.86% of miRNA-disease pairs known to be associated. This reduction made NCPMDA not as performative as presented in Gu *et al*.’s study. In addition, it is worth noting that MiRAI had a low AUC of only 0.6299, worse than the AUC of 0.867 presented in Pasquier *et al*.’ literature [50], because the model was based on collaborative filtering that is known to have the data sparsity problem. The training dataset in our study was sparse, where the average number of miRNAs associated with a disease was 14, while the dataset in Pasquier *et al*.’ study included 83 diseases with at least 20 known associated miRNAs. Evaluated using a sparser dataset, MiRAI became less performative. We believe that using our dataset to assess models would be a more realistic evaluation than using Pasquier *et al*.’s dataset, because the relatedness between miRNAs and diseases remains mostly unknown—currently the biological datasets available to research have a just small amount of labeled data and a large amount of unlabeled data. Our method overcame the data sparsity problem and could be applied to this kind of datasets to make effective predictions.

To evaluate LRSSLMDA’s performance variance, we further carried out 5-fold cross validation on the same dataset as that in global and local LOOCV. Since 5-fold cross validation was a global evaluation, MiRAI and RWRMDA were not included in this comparison. The 5430 known miRNA-disease associations were randomly divided into five subsets with an equal size. Each subset was regarded as the test samples in turn and the rest four were used as the training samples. Again, the miRNA-disease pairs without known association evidences were considered as candidates and we recorded the rank of each test sample against them. Finally, an ROC was produced to calculate the AUC. We repeated this procedure for 100 times to achieve a sound estimate of the average prediction accuracy of LRSSLMDA and obtained an AUC of 0.9181+/-0.0004, surpassing that for PBMDA (0.9172+/-0.0007), MCMDA (0.8767+/-0.0011), MaxFlow (0.8579+/-0.0010), NCPMDA (0.8763+/-0.0008), WBSMDA (0.8185+/-0.0009), RLSMDA (0.8569+/-0.0020) and HDMP (0.8342+/-0.0010). Moreover, the AUC’s standard deviation of 0.0004 was one-fifth of that for RLSMDA (0.0020) and about one-third of that for MCMDA (0.0011), and was also noticeably less than that for the remaining five models. This means that, in addition to its superior prediction power, LRSSLMDA was also a stable model with a lower performance variance than others. Another observation was that the average AUC of 0.8763 for NCPMDA was noticeably lower than its AUC of 0.9073 in global LOOCV. In contrast, for all other models, the two values were very similar to each other. This observation again proved the sensitivity of NCPMDA to the percentage of known associations in the training dataset. In global LOOCV 5429/(495×383) = 2.86% of all miRNA-disease pairs in the training dataset were associated, while in 5-fold cross validation 4344/(495×383) = 2.29% of all miRNA-disease pairs in the training dataset were associated. Again, this reduction in percentage impaired NCPMDA’s prediction accuracy.

According to the above comparison, and to our knowledge, LRSSLMDA was by far the most performative machine learning-based model for miRNA-disease association prediction, whereas PBMDA and NCPMDA were the most state-of-the-art network analysis-based models, though there existed a high risk that NCPMDA was sensitive to the percentage of known miRNA-disease associations and would not perform as well with different datasets. Furthermore, it is worth mentioning that the dimensionality reduction technique used in LRSSLMDA facilitated its extendibility to high dimensional datasets. Therefore, the model’s superiority over other models would likely become even more significant in the future with the availability of more feature profiles for miRNAs/diseases as a result of continuous research.

Finally, to assess the predictability of the statistical feature profile and the graph theoretical profile in our study, we used each profile separately for prediction in the above-mentioned three cross validation schemes. Table 1 records the corresponding AUCs and the AUCs for LRSSLMDA with both profiles used. In global LOOCV, the graph theoretical profile achieved a slightly higher predictive accuracy (an AUC of 0.9174) than the statistical profile (with an AUC of 0.9171). This indicated that the former profile was more advantageous in simultaneously uncovering novel miRNA-disease associations for all diseases than the latter. But in local LOOCV, the statistical profile (with an AUC of 0.8405) became superior to the graph theoretical profile (with an AUC of 0.8375), implying that the former would outperform the latter when making predictions for a specific disease. In 5-fold cross validation, like global LOOCV, the graph theoretical profile (with an average AUC of 0.9177) performed better than the statistical profile (with an average AUC of 0.9174), although both of them had an equally low standard deviation of 0.0004. Overall, using either of the two profiles alone for prediction would yield a satisfactory performance; however, only by involving both profiles could our model achieve the best possible predictive performance, that is, an AUC of 0.9178 in global LOOCV, an AUC of 0.8418 in local LOOCV and an average AUC of 0.9181+/-0.0004 in 5-fold cross validation.

**Table 1:** To evaluate the predictability of different feature profiles in our study, the statistical profile and the graph theoretical profile were used separately for prediction in global LOOCV, local LOOCV and 5-fold cross validation. The corresponding AUCs are shown in the second and third columns, and compared with the AUCs for LRSSLMDA with both profiles in the fourth column.

Experimental results	LRSSLMDA with statistical profile only	LRSSLMDA with graph theoretical profile only	LRSSLMDA with both profiles
AUC in global LOOCV	0.9171	0.9174	0.9178
AUC in local LOOCV	0.8405	0.8375	0.8418
average AUC in 5-fold cross validation	0.9174+/-0.0004	0.9177+/-0.0004	0.9181+/-0.0004

### Case studies

Three types of case studies on five important human diseases were carried out to demonstrate the predictive power of LRSSLMDA. The first type concerned with Colon Neoplasms, Lymphoma and Kidney Neoplasms. The known miRNA-disease associations in HMDD v2.0 were used as the training dataset for the model. For each investigated disease, candidate miRNAs were ranked in terms of their predicted association scores. Then, the top 50 candidates were validated by 1) two other prominent miRNA-disease association databases, namely, dbDEMC [58] and miR2Disease [59], and 2) more recent experimental literatures. As a result of inner joining the three databases, 232 of the 5430 known miRNA-disease associations in HMDD v2.0 also existed in miR2Disease, and 546 associations also existed in dbDEMC. Despite this, there was no overlap between the training samples and the prediction lists. This was because in case studies only candidate miRNAs for an investigated disease were ranked and confirmed by experimental evidences. As has been defined, a candidate miRNA was a miRNA unassociated with the investigated disease according to HMDD v2.0. Therefore, none of the top 50 predictions existed in HMDD v2.0 and validation of the predictions was completely independent of this training database. To facilitate further experimental validations, we used LRSSLMDA to produce a complete prediction list for all the 383 diseases in HMDD v2.0 (See S1 Table). In the second type of case study, we sought to demonstrate the model’s applicability to diseases with no known associated miRNAs and used Esophageal Neoplasms as an example. All the known miRNAs related to this cancer were removed from the training samples so that prioritizing candidate miRNAs would only depend on the information of other diseases’ known associated miRNAs and the similarity information of diseases and miRNAs. In this case study only, we built our model solely from the disease perspective, since the investigated disease was made to have no known associated miRNAs. In the third type of case study, the model was trained by 1395 known miRNA-disease associations between 271 miRNAs and 137 diseases from the old version of HMDD, that is, HMDD v1.0. Breast Neoplasms was the investigated disease and its predicted miRNAs were validated against databases including HMDD v2.0, dbDEMC and miR2Disease as well as more recent studies. We implemented this case study to illustrate the applicability of LRSSLMDA to different datasets other than that in HMDD v2.0. The results for the five cancers in the three types of case studies are listed as follows.

Colon Neoplasms (CN) is a cancer arising from the colon or rectum of humans and is more commonly found in developed countries than developing ones [60]. According to the most recent statistics [61], 135,430 newly diagnosed CN cases and 50,260 deaths caused by this disease are expected in the United States in 2017. Both the CN incidence and mortality rates experienced a continuous decline over the past several decades, partly because of the introduction and wide adoption of screening tests [62]. Nowadays, the screening technology could be improved by the utilization of miRNAs as new biomarkers [63,64]. Studies have shown that miR-126 and miR-145 suppress the CN cell growth via targeting the phosphatidylinositol 3-kinase signaling and the insulin receptor substrate-1, respectively [65,66]. We used LRSSLMDA to uncover more CN-related miRNAs and confirmed 43 out of the top 50 potential miRNAs based on dbDEMC and miR2Disease. Among the remaining seven predictions, six were validated by more recent studies: miR-92a was determined to directly target the anti-apoptosis molecule BCL-2-interacting mediator of cell death (BIM) in CN tissues and an anti-miR-92a antagomir led to the apoptosis of CN cell lines [67]; overexpressed miR-199a-3p (the 3p arm of the pre-miRNA for miR-199a) contributed to the late TNM stage in CN and transfecting miR-199a-3p inhibitor into CN SW480 cells could significantly limit the cell proliferation [68]; miR-142-3p (the 3p arm of the pre-miRNA for miR-142) functioned as a CN suppressor through targeting CD133, leucine-rich-repeat-containing G-protein-coupled receptor 5 (Lgr5) and ATP binding cassette (ABCG2) [69]; miR-146b enhanced the proliferation of CN by targeting the calcium-sensing receptor (CaSR) and impairing the anti-proliferative and pro-differentiating actions of calcium [70]; miR-150 was found to be a tumor suppressor in CN by targeting c-Myb [71]; overexpressed miR-122 and its concomitantly suppressed target gene, cationic amino acid transporter 1 (CAT1), would contribute to the development of CN liver metastasis [72]. Overall, combining the above experimental evidences gave a confirmation of 49 out of the top 50 potential miRNAs (See Table 2).

**Table 2:** Prediction of the top 50 potential Colon Neoplasms-related miRNAs based on known associations in HMDD v2.0 database. The first column records top 1–25 related miRNAs. The third column records the top 26–50 related miRNAs. The evidences for the associations were either dbDEMC and miR2Disease or more recent experimental literatures with the corresponding PMIDs.

miRNA	evidence	miRNA	evidence
hsa-mir-21	dbDEMC;miR2Disease	hsa-mir-210	dbDEMC
hsa-mir-155	dbDEMC;miR2Disease	hsa-mir-199a	23292866
hsa-mir-146a	dbDEMC	hsa-mir-181a	dbDEMC;miR2Disease
hsa-mir-125b	dbDEMC	hsa-mir-200a	unconfirmed
hsa-mir-34a	dbDEMC;miR2Disease	hsa-mir-133a	dbDEMC;miR2Disease
hsa-mir-20a	dbDEMC;miR2Disease	hsa-mir-34c	miR2Disease
hsa-mir-221	dbDEMC;miR2Disease	hsa-mir-9	dbDEMC;miR2Disease
hsa-mir-16	dbDEMC	hsa-mir-142	23619912
hsa-mir-92a	21883694	hsa-let-7c	dbDEMC
hsa-mir-18a	dbDEMC;miR2Disease	hsa-mir-146b	26178670
hsa-mir-19b	dbDEMC;miR2Disease	hsa-mir-106b	dbDEMC;miR2Disease
hsa-mir-29a	dbDEMC;miR2Disease	hsa-mir-181b	dbDEMC;miR2Disease
hsa-mir-19a	dbDEMC;miR2Disease	hsa-mir-182	dbDEMC;miR2Disease
hsa-let-7a	dbDEMC;miR2Disease	hsa-mir-150	25230975
hsa-mir-143	dbDEMC;miR2Disease	hsa-mir-133b	dbDEMC;miR2Disease
hsa-mir-1	dbDEMC;miR2Disease	hsa-mir-203	dbDEMC;miR2Disease
hsa-mir-15a	dbDEMC	hsa-let-7d	dbDEMC
hsa-mir-29b	dbDEMC;miR2Disease	hsa-mir-196a	dbDEMC;miR2Disease
hsa-mir-223	dbDEMC;miR2Disease	hsa-let-7e	dbDEMC
hsa-mir-200b	dbDEMC	hsa-mir-30a	miR2Disease
hsa-mir-222	dbDEMC	hsa-mir-148a	dbDEMC
hsa-mir-31	dbDEMC;miR2Disease	hsa-mir-141	dbDEMC;miR2Disease
hsa-mir-200c	dbDEMC;miR2Disease	hsa-mir-122	23373973
hsa-mir-29c	dbDEMC	hsa-mir-124	dbDEMC
hsa-let-7b	dbDEMC;miR2Disease	hsa-mir-214	dbDEMC

Lymphoma is the most common cancer in adolescents, accounting for 21% of all the cancer cases [61]. Across all age groups, 80,500 new lymphoma incidences and 20,140 mortalities due to the cancer are expected in the United States in 2017 [61]. There are many types of lymphomas but broadly they fall into Hodgkin Lymphoma (HL) or non-Hodgkin Lymphoma (NHL). Experiments have shown that miR-494, miR-1973 and miR-21 could not only be used as diagnostic biomarkers but also circulating cell-free treatment response biomarkers in HL [73]. An example of NHL-miRNA association is that the subtype of NHL, canine B-cell lymphoma, has been found to experience an upregulated expression of miR-19a in the normal lymph nodes [74]. We implemented LRSSLMDA to predict more lymphoma-related miRNAs. Out of the top 50 potential miRNAs, 41 were verified by dbDEMC and miR2Disease; and, among the rest nine predictions, three were confirmed by more recent literatures. MiR-125b-5p (the 5p arm of the pre-miRNA for miR-125b) could upregulate the growth of cutaneous T-cell lymphomas (CTCL) cells, shorten the median survival rate of CTCL patients and promote cellular resistance to proteasome inhibitors by modulating MAD4 proteins [75]. Overexpressed miR-142-5p (the 5p arm of the pre-miRNA for miR-142) was observed in gastric MALT lymphoma, playing a pivotal role in pathogenesis of this cancer [76]. Lastly, the overexpression of miR-146b-5p (the 5p arm of the pre-miRNA for miR-146b) impeded the diffuse large B-cell lymphoma (DLBCL) cell proliferation and this miRNA’s low expression level could predict ineffective treatment response of DLBCL to cyclophosphamide, doxorubicin, vincristine, and prednisone (CHOP) [77]. Consequently, 44 out of the top 50 potential lymphoma-associated miRNAs were proved by experiments (See Table 3).

**Table 3:** Prediction of the top 50 potential Lymphoma-related miRNAs based on known associations in HMDD v2.0 database. The first column records top 1–25 related miRNAs. The third column records the top 26–50 related miRNAs. The evidences for the associations were either dbDEMC and miR2Disease or more recent experimental literatures with the corresponding PMIDs.

miRNA	evidence	miRNA	evidence
hsa-mir-125b	23527180	hsa-mir-451a	unconfirmed
hsa-mir-34a	dbDEMC	hsa-mir-103a	unconfirmed
hsa-mir-221	dbDEMC	hsa-mir-195	dbDEMC
hsa-mir-145	dbDEMC	hsa-mir-30a	dbDEMC
hsa-mir-29a	dbDEMC	hsa-let-7i	dbDEMC
hsa-mir-29b	dbDEMC	hsa-mir-378a	unconfirmed
hsa-mir-143	dbDEMC	hsa-mir-205	dbDEMC
hsa-mir-1	dbDEMC	hsa-mir-96	dbDEMC
hsa-let-7a	dbDEMC	hsa-mir-214	dbDEMC
hsa-mir-222	dbDEMC	hsa-mir-196a	dbDEMC
hsa-mir-223	dbDEMC	hsa-let-7f	dbDEMC
hsa-mir-199a	dbDEMC	hsa-mir-7	dbDEMC
hsa-mir-31	dbDEMC	hsa-mir-183	dbDEMC
hsa-let-7b	dbDEMC	hsa-mir-34b	dbDEMC
hsa-mir-142	23209550	hsa-let-7g	dbDEMC
hsa-mir-181b	dbDEMC	hsa-mir-100	dbDEMC
hsa-let-7c	dbDEMC	hsa-mir-148a	dbDEMC
hsa-mir-146b	24931464	hsa-mir-141	dbDEMC
hsa-mir-34c	unconfirmed	hsa-mir-193a	unconfirmed
hsa-mir-133a	dbDEMC	hsa-mir-15b	dbDEMC
hsa-mir-106b	dbDEMC	hsa-mir-27a	dbDEMC
hsa-mir-9	dbDEMC	hsa-mir-10b	dbDEMC
hsa-let-7e	dbDEMC	hsa-mir-106a	dbDEMC
hsa-let-7d	dbDEMC	hsa-mir-375	unconfirmed
hsa-mir-182	dbDEMC	hsa-mir-93	dbDEMC

Kidney Neoplasms (KN) constitutes about 3.8% of all new cancer cases [78] and so is a less common cancer compared with CN and lymphoma. It has been estimated that in 2017 the United States will witness 63,990 new KN cases and 14,400 deaths due to KN [61]. Renal cell carcinoma (RCC) accounts for nearly 80–85% of KN tumors [79] and its diagnosis was made easier by the application of imaging methods such as ultrasound and abdominal CT with or without pelvic CT [80,81]. MiRNAs hold the potential of being novel biological diagnostic targets for KN. For example, a systematic review [82] has reported the down-expression of miR-141 and miR-200 and the up-expression of miR-23b, miR-29b and miR-438-3p in RCCs. We used LRSSLMDA to discover more KN-related miRNAs. Out of the top 50 candidates, 41 were confirmed by dbDEMC and miR2Disease, while seven other candidates were verified by more recent studies as follows: a lately study [83] revealed that down-regulated miR-125b could inhibit the RCC cell migration and invasion, and result in cell apoptosis, though it had no observed impact on the RCC cell proliferation; miR-221 could promote clear cell RCC (ccRCC) proliferation, migration and invasion via directly inhibiting the tumor suppressor TIMP2 [84]; an inverse correlation between the Von Hippel-Lindau (VHL) gene expression and miR-92a was found in ccRCC patients in the study [85], suggesting this miRNA’s oncogenic role in the tumorigenesis of ccRCC; let-7b was considerably under-expressed in ccRCC tissues and its dysregulation was associated with the pathological grade of ccRCC [86]; a low expression of both miR-133a and miR-1 could up-regulate the oncogenic luciferase assay revealed transgelin-2 (TAGLN2), contributing to the development of RCC [87]; oncogene miR-142-3p (the 3p arm of the pre-miRNA for miR-142) was significantly more overexpressed in RCC tissues than adjacent normal tissues and down-regulated miRNA could induce the apoptosis in RCC 786-O and ACHN cells [88]; miR-30a-5p (the 5p arm of the pre-miRNA for miR-30a) experienced considerably downregulation in RCC tissues and cells [89]. As a result, 48 out of the top 50 potential KN-related miRNAs were confirmed by biological evidences (See Table 4).

**Table 4:** Prediction of the top 50 potential Kidney Neoplasms-related miRNAs based on known associations in HMDD v2.0 database. The first column records top 1–25 related miRNAs. The third column records the top 26–50 related miRNAs. The evidences for the associations were either dbDEMC and miR2Disease or more recent experimental literatures with the corresponding PMIDs.

miRNA	evidence	miRNA	evidence
hsa-mir-155	dbDEMC	hsa-mir-199a	dbDEMC;miR2Disease
hsa-mir-146a	dbDEMC	hsa-mir-29c	dbDEMC;miR2Disease
hsa-mir-17	miR2Disease	hsa-mir-181a	dbDEMC
hsa-mir-125b	28599452	hsa-mir-200a	dbDEMC
hsa-mir-20a	dbDEMC;miR2Disease	hsa-mir-133a	21745735
hsa-mir-34a	dbDEMC	hsa-mir-142	28559989
hsa-mir-145	dbDEMC	hsa-mir-34c	dbDEMC
hsa-mir-221	26191221	hsa-let-7c	dbDEMC
hsa-mir-16	dbDEMC	hsa-mir-9	dbDEMC
hsa-mir-126	dbDEMC;miR2Disease	hsa-mir-150	dbDEMC;miR2Disease
hsa-mir-92a	22043236	hsa-mir-146b	dbDEMC
hsa-mir-18a	dbDEMC	hsa-mir-182	dbDEMC;miR2Disease
hsa-mir-19b	dbDEMC;miR2Disease	hsa-mir-106b	dbDEMC;miR2Disease
hsa-mir-29a	dbDEMC;miR2Disease	hsa-mir-181b	dbDEMC
hsa-let-7a	dbDEMC	hsa-mir-203	dbDEMC
hsa-mir-1	dbDEMC	hsa-mir-133b	unconfirmed
hsa-mir-19a	dbDEMC	hsa-let-7e	unconfirmed
hsa-mir-143	dbDEMC	hsa-mir-30a	27035333
hsa-mir-29b	dbDEMC;miR2Disease	hsa-let-7d	dbDEMC
hsa-mir-223	dbDEMC	hsa-mir-148a	dbDEMC
hsa-mir-31	dbDEMC	hsa-mir-196a	dbDEMC
hsa-mir-200b	dbDEMC;miR2Disease	hsa-mir-214	dbDEMC;miR2Disease
hsa-mir-222	dbDEMC	hsa-mir-7	dbDEMC;miR2Disease
hsa-mir-210	dbDEMC;miR2Disease	hsa-mir-34b	dbDEMC
hsa-let-7b	25951903	hsa-mir-124	dbDEMC

Esophageal Neoplasms (EN) is a cancer developed from the esophagus and ranks sixth among all cancers in terms of mortality [90]. I the United States, for both sexes the total estimated new EN cases will be 16,940 in 2017, while the total projected death caused by EN will be 15,690 [61]. Population-based screening for EN was not viable due to the relatively low incidence, the absence of early symptoms and the rarity of a hereditary form of the cancer [90,91]. Fortunately, monitoring miRNA expression may be useful for detecting EN. Experiments have indicated that expression profiles of mir-203, mir-205 and mir-21 can determine esophageal tumor histology and discriminate normal tissues from tumorous ones [92]. We trained LRSSLMDA to uncover more EN-related miRNAs and illustrate our model’s applicability to diseases without known associated miRNAs. Out of the top 50 predictions, 49 were confirmed by dbDEMC and miR2Disease (See Table 5). The remaining candidate, mir-122, was found to assist Tanshinone IIA in inhibiting EN cell growth [93]. In addition, miRNA response elements (MREs) of miR-122 and mir-144 employed in EN patients would induce EN cell apoptosis while preserving normal cells [94]. However, whether a direct link exists between miR-122 and EN deserves further investigation.

**Table 5:** Prediction of the top 50 potential Esophageal Neoplasms-related miRNAs based on known associations in HMDD v2.0 database. All the known miRNAs related to this cancer were removed from the training samples, and LRSSLMDA was built solely from the disease perspective. The first column records top 1–25 related miRNAs. The third column records the top 26–50 related miRNAs. The evidences for the associations were dbDEMC, miR2Disease and HMDD v2.0.

miRNA	evidence	miRNA	evidence
hsa-mir-21	dbDEMC;miR2Disease;HMDD v2.0	hsa-mir-181a	dbDEMC
hsa-mir-155	dbDEMC;HMDD v2.0	hsa-mir-133a	dbDEMC;HMDD v2.0
hsa-mir-146a	dbDEMC;HMDD v2.0	hsa-mir-31	dbDEMC;HMDD v2.0
hsa-mir-17	dbDEMC	hsa-mir-29c	dbDEMC;HMDD v2.0
hsa-mir-125b	dbDEMC	hsa-let-7b	dbDEMC;HMDD v2.0
hsa-mir-34a	dbDEMC;HMDD v2.0	hsa-mir-210	dbDEMC;HMDD v2.0
hsa-mir-20a	dbDEMC;HMDD v2.0	hsa-mir-200c	dbDEMC;HMDD v2.0
hsa-mir-145	dbDEMC;HMDD v2.0	hsa-mir-150	dbDEMC;HMDD v2.0
hsa-mir-221	dbDEMC	hsa-mir-142	dbDEMC
hsa-mir-16	dbDEMC	hsa-mir-146b	dbDEMC
hsa-mir-29a	dbDEMC	hsa-let-7c	dbDEMC;HMDD v2.0
hsa-mir-92a	HMDD v2.0	hsa-mir-182	dbDEMC
hsa-mir-19b	dbDEMC	hsa-mir-106b	dbDEMC
hsa-mir-18a	dbDEMC	hsa-mir-34c	dbDEMC;HMDD v2.0
hsa-mir-126	dbDEMC;HMDD v2.0	hsa-mir-200a	dbDEMC;HMDD v2.0
hsa-mir-1	dbDEMC	hsa-mir-122	unconfirmed
hsa-mir-29b	dbDEMC	hsa-mir-9	dbDEMC
hsa-mir-19a	dbDEMC;HMDD v2.0	hsa-mir-181b	dbDEMC
hsa-let-7a	dbDEMC;HMDD v2.0	hsa-mir-133b	dbDEMC
hsa-mir-15a	dbDEMC;HMDD v2.0	hsa-let-7e	dbDEMC
hsa-mir-143	dbDEMC;HMDD v2.0	hsa-mir-195	dbDEMC
hsa-mir-222	dbDEMC	hsa-mir-30a	dbDEMC
hsa-mir-223	dbDEMC;miR2Disease;HMDD v2.0	hsa-let-7d	dbDEMC
hsa-mir-200b	dbDEMC	hsa-mir-148a	dbDEMC;HMDD v2.0
hsa-mir-199a	dbDEMC;HMDD v2.0	hsa-mir-196a	dbDEMC;miR2Disease;HMDD v2.0

Breast Neoplasms (BN) is a common cancer in developed countries. In the United States, for instance, one in eight of its population has acquired BN [95] and in 2017 there will be approximately 63,410 newly diagnosed cases [61]. The detection methods for BN mainly include clinical breast examination for earlier-stage cancers and mammography is recommended for women aged over 40 [96]. Curing BN is highly possible given an early stage diagnosis, which could be achieved by involving easily accessible and sensitive miRNAs [97]. MiRNA dysregulations exist in BN patients through polymorphisms in the sequence of the miRNA, its binding sites in target genes, or through epigenetic mechanisms [98]. An example is the elevated expression level of miR-195 which occurred exclusively in BN patients and could be used to differentiate BN from other Malignancies [99]. We trained LRSSLMDA by known miRNA-disease association data from HMDD v1.0. The HMDD v2.0, dbDEMC and miR2Disease databases confirmed 47 out of the top 50 potential BN-related miRNAs, while more recent experimental literatures verified two of the rest three ones. MiR-494 could suppress the progression of BN in vitro by targeting CXCR4 through the Wnt/β-catenin signaling pathway [100]; and the expression level of miR-30e was lowered in both plasma and breast cancer tissues of BN patients and plasma miR-30e expression was statistically related to the patients age and clinical stage of BN [101]. To conclude, experimental evidences from databases and other publications validated 49 out of the top 50 potential BN-associated miRNAs (See Table 6).

**Table 6:** Prediction of the top 50 potential Breast Neoplasms-related miRNAs based on known associations in the old version of HMDD, that is, HMDD v1.0. The first column records top 1–25 related miRNAs. The third column records the top 26–50 related miRNAs. The evidences for the associations were either HMDD v2.0, dbDEMC and miR2Disease or more recent experimental literatures with the corresponding PMIDs.

miRNA	evidence	miRNA	evidence
hsa-mir-659	dbDEMC	hsa-mir-191	dbDEMC;miR2Disease;HMDD v2.0
hsa-let-7e	dbDEMC;HMDD v2.0	hsa-mir-192	dbDEMC
hsa-let-7c	dbDEMC;HMDD v2.0	hsa-mir-129	dbDEMC;HMDD v2.0
hsa-let-7b	dbDEMC;HMDD v2.0	hsa-mir-99b	dbDEMC
hsa-let-7i	dbDEMC;miR2Disease;HMDD v2.0	hsa-mir-199b	dbDEMC;HMDD v2.0
hsa-mir-16	dbDEMC;HMDD v2.0	hsa-mir-195	dbDEMC;miR2Disease;HMDD v2.0
hsa-mir-92a	HMDD v2.0	hsa-mir-494	25955111
hsa-mir-130b	dbDEMC	hsa-mir-299	dbDEMC;HMDD v2.0
hsa-mir-27a	dbDEMC;miR2Disease;HMDD v2.0	hsa-mir-148a	dbDEMC;miR2Disease;HMDD v2.0
hsa-mir-126	dbDEMC;miR2Disease;HMDD v2.0	hsa-mir-26a	dbDEMC;miR2Disease;HMDD v2.0
hsa-let-7g	dbDEMC;HMDD v2.0	hsa-mir-30e	27012041
hsa-mir-373	dbDEMC;miR2Disease;HMDD v2.0	hsa-mir-101	dbDEMC;miR2Disease;HMDD v2.0
hsa-mir-30a	miR2Disease;HMDD v2.0	hsa-mir-135a	dbDEMC;HMDD v2.0
hsa-mir-223	dbDEMC;HMDD v2.0	hsa-mir-365	miR2Disease
hsa-mir-372	dbDEMC	hsa-mir-107	dbDEMC;HMDD v2.0
hsa-mir-500	unconfirmed	hsa-mir-497	dbDEMC;miR2Disease;HMDD v2.0
hsa-mir-423	HMDD v2.0	hsa-mir-181a	dbDEMC;miR2Disease;HMDD v2.0
hsa-mir-106a	dbDEMC	hsa-mir-24	dbDEMC;HMDD v2.0
hsa-mir-381	dbDEMC	hsa-mir-18b	dbDEMC;HMDD v2.0
hsa-mir-432	dbDEMC	hsa-mir-29c	dbDEMC;miR2Disease;HMDD v2.0
hsa-mir-130a	dbDEMC	hsa-mir-452	dbDEMC;HMDD v2.0
hsa-mir-520b	dbDEMC;HMDD v2.0	hsa-mir-100	dbDEMC;HMDD v2.0
hsa-mir-32	dbDEMC	hsa-mir-182	dbDEMC;miR2Disease;HMDD v2.0
hsa-mir-98	dbDEMC;miR2Disease	hsa-mir-411	dbDEMC;HMDD v2.0
hsa-mir-28	dbDEMC	hsa-mir-22	dbDEMC;miR2Disease;HMDD v2.0

## Discussion

The clinical significance of uncovering disease-associated miRNAs lies in their potential roles of therapeutic targets and diagnostic biomarkers for diseases. We introduced a novel computational model for predicting disease-miRNA associations by Laplacian regularized sparse subspace learning (LRSSLMDA). It would effectively complement to existing experimental methods in a way that the candidate miRNAs would be initially prioritized based on available biological data, followed by experimental validations on the most promising candidates. LRSSLMDA was developed as follows. The first step was Data Preparation. The Gaussian interaction profile kernel similarity scores for miRNAs and diseases were calculated from known miRNA-disease associations. Then we constructed the integrated similarity for miRNAs and diseases. In addition, statistical features and graph theoretic features for miRNAs and diseases were extracted from the integrated similarity. The second step was Model Formation. From the respective miRNA/disease perspective, we built an objective function from the common miRNA/disease subspace for the miRNA/disease feature spaces, an *L*_1_-norm constraint and Laplacian regularization terms. This step resulted in two objective functions: one from the view of miRNAs and the other from the view of diseases. The third step was Optimization where we optimized the objective functions and lastly combined the optimization results to attain the final prediction outcomes. Albeit inspired by Liang *et al*.’s method, our model had a substantial innovation: less input data was needed for prediction without sacrificing the predictive performance; disease-related feature profiles were efficiently exploited; and the model could effectively prioritize candidate miRNAs for diseases without known associated miRNAs. Cross validations were carried out to assess the prediction performance of LRSSLMDA. Impressively, it outperformed ten previous models (MCMDA, HGIMDA, WBSMDA, HDMP, RLSMDA and RWRMDA) under the global and local LOOCV frameworks and its prediction stability was reflected by a low standard deviation in results of the 5-fold cross validation. To our knowledge, LRSSLMDA is one of the very few models that achieved an AUC greater than 0.9 in global LOOCV. In addition, three types of case studies on five diseases demonstrated LRSSLMDA’s prediction accuracy. For each disease, a majority of the top 50 potential related miRNAs were confirmed by experimental literatures.

The reliable performance of LRSSMDA stemmed from four factors. First, comprehensive statistical features and graph theoretic features were constructed from the integrated similarity matrices for miRNAs and diseases. The statistical profile included the mean, the sum, the quantiles and the histogram distributions of the similarity scores, while the graph theoretic profile recorded the neighbor count, the centrality measures and Page-Rank scores of the network graphs built from the integrated similarity matrices for miRNAs and diseases. Moreover, because these two feature profiles made full use of the miRNA similarity and the disease similarity, and because functionally similar miRNAs tend to be related to phenotypically similar diseases [31–33], our model could effectively uncover miRNAs associated with diseases that had no known associated miRNAs. This was demonstrated in the fourth case study on Esophageal Neoplasms, where 49 out of the top 50 predictions were confirmed by experimental literatures. Second, dimensionality reduction was implemented via projecting the profiles to a common subspace, which removed the multi-collinearity in them. LRSSLMDA sought to determine the most useful features for differently profiles simultaneously. Third, Laplacian regularization was used to keep the local structure of the feature spaces; it also captured the similarities between known miRNA-related diseases and between known disease-related miRNAs. This resonated with the assumption that functionally similar miRNAs tend to be related to semantically similar diseases. Fourth, the sparse feature selection facilitated by *L*_1_-norm assigned higher weights to the most useful features, further improving the performance of LRSSLMDA.

However, there is noticeable room for improvement in LRSSLMDA. The miRNA and disease similarity calculations presented in this study might not be the perfect methods and we expect more biological information to be incorporated into the calculations in the future to fine-tune the similarity measures. In addition, by far the known miRNA-disease associations have a large degree of sparsity (with only 2.86% of 189,585 miRNA-disease pairs being labeled). Accumulating experimental evidences will confirm more associations that would diminish the prediction bias of LRSSLMDA. As a final point, the increasing understanding towards miRNAs and diseases would eventually facilitate a miRNA-disease association prediction that not solely depends on miRNAs’ functional similarity and diseases’ semantic similarity, but also other possible miRNA and disease profiles. Adding new profiles into LRSSLMDA would lead to a more comprehensive analysis and hopefully an improved accuracy of miRNA-disease association prediction. Therefore, we believe that our model would perform even better in future research.

## Supporting information

S1 Table We applied LRSSMDA to prioritize all the candidate miRNA-disease pairs based on all the known miRNA-disease associations in HMDD ver2.0 database as training samples. This prediction result is released for further experimental validation and research.
(XLSX)

S2 Table The human miRNA-disease associations dataset used to train LRSSLMDA was retrieved from the latest version of the HMDD database, covering 5430 experimentally confirmed associations between 495 miRNAs and 383 diseases. (XLSX)
